# Efficacy and safety of 2-hour urokinase regime in acute pulmonary embolism: a randomized controlled trial

**DOI:** 10.1186/1465-9921-10-128

**Published:** 2009-12-29

**Authors:** Chen Wang, Zhenguo Zhai, Yuanhua Yang, Yadong Yuan, Zhaozhong Cheng, Lirong Liang, Huaping Dai, Kewu Huang, Weixuan Lu, Zhonghe Zhang, Xiansheng Cheng, Ying H Shen, China Venous Thromboembolism Study Group (CVTESG)

**Affiliations:** 1 Beijing Institute of Respiratory Medicine, Beijing Chao-Yang Hospital, Capital Medical University, Beijing, PR China; 2 The Second Affiliated Hospital of Hebei Medical University, Hebei, PR China; 3 The Affiliated Hospital of Medical College of Qingdao, Shandong, PR China; 4 Peking Union Medical College Hospital, Chinese Academy of Medical Sciences, Beijing, PR China; 5 The Affiliated Hospital of Dalian Medical University, Liaoning, PR China; 6 Beijing Fuwai Hospital, Chinese Academy of Medical Sciences, Beijing, PR China; 7 Baylor College of Medicine, Houston, Texas, USA

## Abstract

**Backgrounds:**

Urokinase (UK) 2 200 U/kg·h for 12 hours infusion(UK-12 h)is an ACCP recommended regimen in treating acute pulmonary embolism (PE). It is unclear whether this dose and time can be reduced further. We compared the efficacy and safety of 20, 000 U/kg for 2 hours (UK-2 h) with the UK-12 h regime in selected PE patients.

**Methods:**

A randomized trial involving 129 patients was conducted. Patients with acute PE were randomly assigned to receive either UK-12 h (n = 70), or UK-2 h (n = 59). The efficacy was determined by the improvement of right heart dysfunction and perfusion defect at 24 h and 14 d post UK treatment. The bleeding incidence, death rate and PE recurrence were also evaluated.

**Results:**

Similarly significant improvements in right heart dysfunction and lung perfusion defects were observed in both groups. Overall bleeding incidents were low in both groups. Major bleeding directly associated with UK infusion occurred in one patient in the UK-2 h group and one in the UK-12 h group. Mortality rates were low, with one reported fatal recurrent in the UK-12 h group and none in the UK-2 h group. When the rate of bleeding, death and PE recurrence were compared separately in the hemodynamic instability and the massive anatomic obstruction subgroups, no significant difference was found.

**Conclusions:**

The UK-2 h regimen exhibits similar efficacy and safety as the UK-12 h regimen for acute PE.

**Trial Registration:**

Clinical trial registered with http://clinicaltrials.gov/ct2/show/NCT00799968 (Identifier: NCT 00799968)

## Background

Urokinase (UK) can produce mild and prolonged thrombolytic state with minimal disturbance of blood coagulation. Its effect is highly controllable when administered at body weight adjusted dosages [1,2]. Additionally, UK is much cheaper than tissue plasminogen activator (rt-PA). The reliable efficacy and safety, as well as the low cost have made UK a valuable agent in pulmonary embolism (PE) thrombolytic therapy, and it is widely used in developing countries.

Currently, no single UK regimen has been shown to be superior over another in treating PE. A loading dose of 4400 IU/kg followed by 2200 IU/kg/hour for 12 hours (UK-12 h) is recommended for acute PE treatment by American College of Chest Physician (ACCP) guideline[3]. However, earlier studies have showed that the bleeding incidence of this regimen was high [4,5]. Reducing infusion time to 2-h have demonstrated promising results with decreased major bleeding rate. Additionally, we have observed that the UK-2 h (20 000 U/kg over 2 h) regime has superior thrombolytic effects over the UK-12 h regimen for fresh thrombi in a canine PE model [6]. Because of the potential advantages of lower bleeding risk, increased effectiveness for fresh thrombi, convenience and lower cost, UK-2 h becomes a promising regimen. However, there is no direct comparison between this regimen with the ACCP recommended UK12 h regimen.

In this study, we compared UK2 h and UK-12 h regimen in treating acute PE in a randomized, controlled, multi-center trial. Acute PE patients with either haemodynamic instability/cardiogenic shock [7,8] or anatomic massive obstruction and right ventricular dysfunction(RVD) were enrolled in the study. Efficacy was assessed by the improvements in right ventricular (RV) function, the pulmonary perfusion on V/Q scan and pulmonary artery obstruction on CT pulmonary angiograms (CTPA) at 24 h and 14 d after treatment. Safety was evaluated by the incidence of bleeding, PE recurrence and death rates.

## Methods

### Study Organization

A prospective, randomized, open label, multi-center trial was conducted between July 2002 and February 2006 in China. The trial was suspended for nearly one year due to the severe acute respiratory syndrome (SARS) outbreak in 2003. A central steering committee was formed in charge of study design, protocol development and standardization, quality control, data verification and analysis. The study protocols were reviewed and approved by institutional boards and ethics committees of all participating centers. Patients enrolled in the study were centrally randomly assigned to the treatment group with a standard computerized randomization program. The clinical information and images of each patient were reviewed independently by the members of the steering committee.

### Patient Selection

Patients aged 18 to 75 years with acute PE (symptoms that occurred within 15 days at time of enrollment) were screened. All diagnoses were confirmed either by a high probability V/Q scan or by the presence of intraluminal filling defect on spiral CTPA. The indication for thrombolytic therapy was: (1) patients with haemodynamic instability or cardiogenic shock; (2) patients with massive pulmonary artery obstruction (obstructions in more than 2 lobes on CTPA, or perfusion defects in more than 7 segments on V/Q scan) and combined right ventricular dysfunction (RVD) and pulmonary hypertension on echocardiographic examination. Studies have shown that these patients are at high risk of fatal conditions and may benefit from thrombolytic therapy[9-11]. Patients were excluded if they had received parenteral heparin for more than 72 hours or had thrombolytic contraindications. Written informed consents were obtained from all the patients.

### Treatment regimens

**Figure 1:**
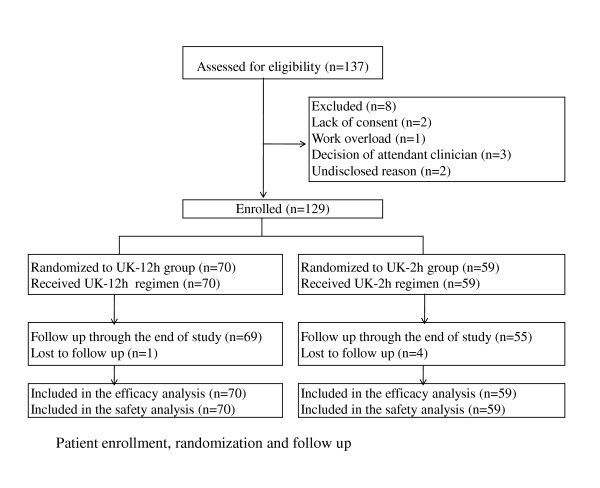
**Study flow diagram**.

Eligible subjects were randomly assigned into either the UK-12 h group (bolus 4 400 U/kg followed by intravenous 2 200 U/kg·h for 12 hours) or UK-2 h group (20 000 U/kg intravenous infusion for 2 hours) (Figure 1). Randomization was performed on a central control manner according to a standard randomization program. UK used in this study was from Guangzhou Techpool (Bio Pharma Co Ltd, China).

After UK infusion, the activated partial thromboplastin time (aPTT) was tested. If the value was less than 80 sec, a subcutaneous injection of low molecular weight heparin (nadroparin, 86 anti-factor Xa IU/kg) was given every 12 hours. Overlapping oral anticoagulant therapy (warfarin) was started on day 1-3 after nadroparin injection to maintain an international normalized ratio (INR) of 2.0 to 3.0. Nadroparin was stopped 4-5 days after warfarin was added and INR was stabilized at 2.0 to 3.0 for at least 2 days. Warfarin was continuously used for at least 3-6 months, and subsequent doses were adjusted to maintain the INR at within 2.0-3.0 range, targeting value 2.5.

### Efficacy

Symptoms and signs of PE in all patients were monitored. Echocardiogram, V/Q lung scan and CTPA were evaluated within 48 hours before UK infusion, and repeated in most patients at 24 h and 14 d after UK treatment. Efficacy was determined by the improvements of right ventricular functions on echocardiogram, the lung perfusion on lung V/Q scans and pulmonary artery obstruction on CTPA.

#### Echocardiograms

Echocardiograms were performed and analyzed by two echocardiographers. Right ventricular function was analyzed by the improvement of (1) right and left ventricular end-diastolic diameter ratio in the parasternal long-axis view (RVED/LVED); (2) right/left ventricular dimension ratio (RV/LV dimension); (3) estimated systolic pulmonary artery pressure (SPAP), in the absence of left ventricular disease or mitral valve disease as previously reported[10,11]. SPAP was measured using the following equation: SPAP was 4 × (V_TR_)^2 ^+ right atrial pressure (RAP). RAP was estimated according to the respiratory motion of IVC.

#### V/Q lung scan

Lung scans were independently reviewed and evaluated by two specialists using the methods described previously [12,13]. Each anatomic segment of the lung was reviewed and the defect within each segment was scored according to the level of perfusion reduction.

#### CTPA

The location and severity of thrombus obstruction of the pulmonary vascular bed were reviewed and evaluated by helical CTPA score system described in previous studies [14,15]. The index is defined as the product N × D, where N is the value of the proximal clot site (equal to the number of segmental branches arising distally) and D is the degree of obstruction. Partial obstruction is scored as 1 and total obstruction is graded as 2.

### Death, bleeding and pulmonary embolism recurrence

Adverse events were monitored for 14 days after initial treatment with UK. Deaths were classified as being due to PE, bleeding, or other causes (including myocardial infarction and unknown causes). Major bleeding included fatal bleeding, intracranial hemorrhage (ICH), or a drop in the hemoglobin concentration by at least 20 g/L, or required transfusion of more than 400 ml red blood cell (within 72 h of initiating UK therapy). Minor bleeding included the bleeding with hemoglobin concentration drop of less than 20 g/L. Recurrence of PE was confirmed by V/Q scanning and/or spiral CTPA. Blood urea nitrogen(BUN) and creatinine(Cr) were monitored during the whole interval of the study. The methods of kidney protection include encouraging patients to drink abundantly or recommending the ward to ensure adequate hydration to patients for 12 hours.

### Statistical analysis

Calculation of sample size demonstrated that 110 patients were required to show a difference of 10 points between treatment groups in percent reduction of the score of CTPA at 24 h from the onset of thrombolysis with 80% power and a two-sided level of significance of 0.05, assuming a standard deviation of 10 points. Data were analyzed according to the intention-to-treat principle.

Categorial data were compared using chi-square test and Fisher's test (2 × 2 table). Comparison of continuous data between the treatment groups were performed using t test or the Willcoxon test as appropriate. Changes in the measurements of Echocardiograms, V/Q scan, and CTPA over time were analyzed using repeated measures analysis of variance. Subgroup analysis was also conducted base on the homodynamic status of massive PE. All reported P values are 2-sided. P < 0.05 was statistically significant. A post hoc subgroup analysis was also conducted base on the homodynamic status of massive PE by using unpaired t test, chi-square test or Fisher's test as appropriate.

## Results

### Patient population and baseline characteristics

137 patients with acute PE were preselected in the participating centers. 125 patients were confirmed with CTPA. 98 patients were evaluated with V/Q at the same time. 8 (7%) were ineligible because of the predefined exclusion criteria. Among the 129 enrolled in this study, 70 were assigned to UK-12 h and 59 were assigned to UK-2 h(Figure 1). There were no significant differences between the two groups in baseline characteristics (Table 1).

**Table 1:** Baseline characteristics

Characteristics	UK-12 h (n = 70)	UK-2 h (n = 59)
**Gender (male/female)**	41/29	37/22
**Age mean ± SD (y)**	55.1 ± 16.1	56.5 ± 14.1
**Weight mean ± SD (kg)**	67.7 ± 11.9	68.2 ± 10.4
**BMI mean ± SD(kg/m2)**	24.4 ± 4.2	24.6 ± 3.5
**Prior DVT or PE, n (%)**	3(4.3)	5(8.6)
**CVD, n (%)**	8(11.4)	7(12.1)
**Hypertension, n (%)**	17(24.3)	18(31.0)
**Diabetes mellitus, n (%)**	6(8.6)	4(6.9)
**COPD, n (%)**	4(5.7)	5(8.6)
**Malignancy, n (%)**	2(2.9)	3(5.2)
**Systolic blood pressure mean ± SD (mmHg)**	127.0 ± 21.0	121.8 ± 17.0
**Diastolic blood pressure mean ± SD (mmHg)**	80.9 ± 12.3	74.2 ± 13.9
**PaO2 (mmHg)**	72.9 ± 20.3	70.8 ± 16.5
**PaCO2 (mmHg)**	34.5 ± 5.9	33.8 ± 6.6
**Respiratory rate mean ± SD (rpm)**	23.1 ± 4.9	23.7 ± 6.4
**Heart rate mean ± SD (bpm)**	96.4 ± 17.6	93.9 ± 17.8
**Hb mean ± SD (g/L)**	128.9 ± 22.3	125.7 ± 20.8
Hemodynamically massive PE*, n (%)	20 (28.6)	20 (33.9)
**Anatomically massive PE with RVD & PH¶, n (%)**	50(71.4)	39 (66.1)

* Arterial hypotension (a systolic arterial pressure <90 mm Hg or a drop in systolic arterial pressure of at least 40 mm Hg for at least 15 minutes) or cardiogenic shock.

¶ Pulmonary artery obstruction > 2 lobes on CTPA or >7 segments on V/Q scan combined with RVD and PH, but without hypotension or cardiogenic shock.

### Efficacy

**Figure 2:**
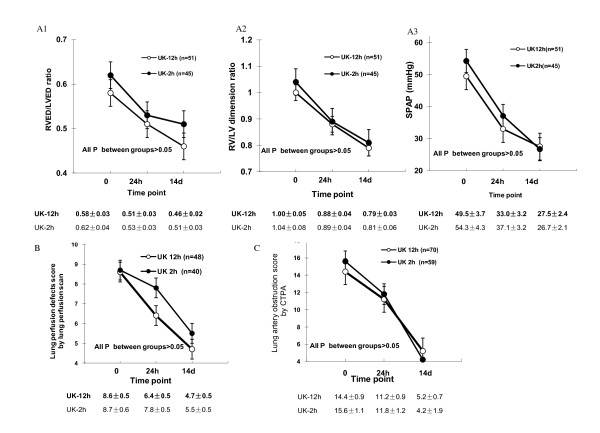
**Comparison of right heart functions (A), lung perfusion defect scores (B) and pulmonary artery obstruction scores (C) between the two treatments (Data are presented with mean ± SE)**.

The improvements of pulmonary artery pressure and RV function were evaluated by echocardiograms. The baseline, 24 h and 14 d follow-up evaluations of RVED/LVED ratio, RV/LV dimension ratio and SPAP were performed in 96 (74%) patients, 51 in the UK-12 h group and 45 in the UK-2 h group. Both treatment groups showed significantly progressive improvements of right ventricular functions determined by all three measurements. No significant difference was found between the two treatment groups in these measurements at different time points (Figure 2A).

Improvement of lung perfusion was determined by V/Q lung scan. The baseline, 24 h and 14 d follow-up V/Q lung scans were conducted in 88 (68%) patients, 48 in the UK-12 h and 40 in the UK-2 h group. Both treatments achieved significant improvements in pulmonary vascular perfusion defects, with similar scores at 24 h and 14 d post rt-PA administration (Figure 2B).

The improvement of pulmonary artery obstruction was assessed by CTPA. 125 patients (96%) underwent baseline, 24 h and 14 d follow up CTPAs, 70 in the UK 12 h group and 55 in the UK 2 h group. Quantitative assessment showed that the two treatment groups had substantial reductions in PE obstructive index at different time points after treatment, with no significant difference between the groups (Figure 2C).

The distributions of significant improvement, slight improvement, no change and worse were similar between the two regimens (Table 2).

**Table 2:** Comparison of the pulmonary artery obstruction improvement scales between the two treatments

	24 hour			14 day		
	
Qualitative Change	UK-12 h (n = 70)	UK-2 h (n = 55)	*P*	UK-12 h (n = 70)	UK-2 h (n = 55)	*P*
**Significant improvement**	34(48.6)	36(61.1)	0.373	47(67.1)	44(74.6)	0.807
**Slight-improvement**	16(22.9)	11(18.6)		17(24.3)	12(20.3)	
**No change**	8(11.4)	8(13.6)		3(4.3)	2(3.4)	
**Worse**	12(17.1)	4(6.8)		3(4.3)	1(1.7)	
Any improvement*	50(71.4)	47(79.7)	0.281	64(91.4)	56(94.9)	0.439

Data presented are number (%) of patients.

The improvements of pulmonary artery obstruction from baseline between the two treatments were compared.

* Any improvement (%) = (significant + slight)/total, which indicates the percentage of overall improvement. Significant improvement- obstruction decreased by ≥ 75%; slight improvement - obstruction decreased by ≥ 25% but < 75%; no change - obstruction reduced by < 25%; worse - obstruction increased in score index.

### Mortality, bleeding, and pulmonary embolism recurrence

The mortality rate was low in both groups. One patient (1.4%) in the UK-12 h group died of recurrent PE. Two (3.4%) patients in the UK-2 h group died, one died at 25 minutes after the initiation of UK due to PE combined with respiratory and congestive heart failure, and the other one died 3 days after the initiation of the treatment due to acute myocardial infarction.

The incidence of overall bleeding in the two groups was similar (13.6% in UK-2 h group vs. 14.3% in UK-12 h group). The incidences of major bleeding and minor bleeding were also similar in the two groups. No intracranial bleeding episode was found in either group. No fatal bleeding episode was reported in either group. It is important to emphasize that the majority of major bleeding occurred 3 days after UK infusion. Only one patient with major bleeding episodes in UK 2 h group and one patient in UK 12 h group were directly associated with UK (occurred with 72 hours after initiation of UK).

One fatal recurrent PE was reported at 4 days post-treatment in the UK-12 h group. No other episode of recurrent symptomatic PE was reported in either group (Table 3).

**Table 3:** Comparison of adverse events during the first 14 days after treatment between the two treatments

Adverse events	UK-12 h(n = 70)	UK-2 h(n = 59)	P
**Death**	1(1.4)	2(3.4)	
**Due to PE**	1(1.4)	1(1.7)	0.462
**Due to Bleeding**	0(0)	0(0)	
**Due to Other Comordities**	0(0)	1(1.7)	
**Recurrent PE**	1(1.4)	0(0)	0.858
**Fatal**	1(1.4)	0(0)	
**Non fatal**	0(0)	0(0)	
**Bleeding complications**	10(14.3)	8(13.6)	0.332
**Major bleeding**	4(5.7)	6(10.2)	
**Fatal bleeding**	0(0)	0(0)	
**Others**	4(5.7)	6(10.2)	
**Minor Bleeding**	6(8.6)	2(3.4)	

Data presented are number (%) of patients.

### Efficacy and safety in patients with hemodynamic instability or massive pulmonary artery obstruction

Further subgroup comparisons were conducted separately in patients with hemodynamic instability and in patients with massive pulmonary artery obstruction. In patient with hemodynamic instability, UK-12 h and UK-2 h produced similar progressive improvements in pulmonary artery obstructions (Table 4). Additionally, no difference was found between these two treatments in terms of death, bleeding, and PE recurrence in these patients. Similarly, these two treatments showed similar efficacy and safety in patients with massive pulmonary vascular obstruction combined with RVD.

**Table 4:** Comparison of efficacy and the adverse events between the two treatments in hemodynamically and anatomically massive PE subgroups

	Hemodynamically massive PE			Anatomically massive PE		
	
	UK 12 h (n = 20)	UK 2 h (n = 20)	p	UK 12 h (n = 50)	UK 2 h (n = 39)	p
**Efficacy**						
Pulmonary artery obstruction score by CTPA*						
**Baseline**	16.1 ± 7.7	17.3 ± 10.5	0.670	13.7 ± 7.2	14.7 ± 7.6	0.523
**24 -hour**	13.1 ± 7.7	13.5 ± 11.7	0.899	10.5 ± 6.9	10.9 ± 8.1	0.783
**14- day**	6.9 ± 7.3	5.2 ± 9.8	0.527	4.5 ± 5.2	3.7 ± 4.6	0.452
**Adverse events**						
**Death**	0 (0)	2 (10.0)	0.487	1(2)****	0(0)	1.000
**Due to PE**	0(0)	1 (5.0)**		0 (0)	0(0)	
**Due to other reason**	0(0)	1 (5.0)***		0 (0)	0(0)	
**Due to Bleeding**	0 (0)	0(0)		0 (0)	0(0)	
**Recurrent PE**	0 (0)	0(0)		1(2)	0(0)	
**Bleeding complications**	3(15)	3(15)	1.000	7(14)	5(13)	1.000
**Major bleeding**	1 (5.0)	3 (15.0)	0.605	3 (6)	3 (7.7)	1.000
**Fatal bleeding**	0 (0)	0(0)		0(0)	0(0)	
**Other**	1 (5.0)	3 (15.0)		3(6)	3 (7.7)	
**Minor bleeding**	2(10.0)	0(0.0)	0.487	4(8)	2(5.1)	0.692

* CT pulmonary angiographic assessment, Data presented are number (%) of patients.**The patient died due to the severity of PE. *** The patient died due to the commodities of acute myocardial infarction. **** The patient died due to recurrent PE.

## Discussion

Our study showed that the UK-2 h regimen exhibited similar efficacy as the UK-12 h regimen in PE thrombolytic therapy, with similar progressive improvements in pulmonary perfusion defects and RVD during the 14 d post-treatment period. The two regimens had similar bleeding incidences and death rates.

### Thrombolytic efficacy

Previous studies have demonstrated that UK12 h and UK 24 h can induce a rapid thrombolysis in pulmonary circulation in acute massive PE [16,17]. Increasing evidence suggests that UK infusion dosage and duration can be reduced further. It has been shown that 3 million IU UK infusion over 2 h followed by heparin therapy is as effective and safe as the rt-PA 100 mg over 2 h regimen [18,19]. A much lower dose of 20 000 IU/kg UK over 2 h has been shown to be effective without severe bleeding and allergic reaction [20]. In this study, we demonstrated that UK-2 h regimen, a much lower dose (20 000 IU/kg) than those used in previous trials (3 million IU/2 h)[18,19], produced similar improvements in pulmonary circulation and right heart dysfunction as the UK-12 h regimen. In our practice, we use thrombolytic therapy instead of anticoagulation therapy alone to treat patients with extensive pulmonary artery obstruction (defined as an obstruction exceeding 50% of the pulmonary vasculature or the occlusion of two or more lobar arteries)[21,22], combined with echocardiographic signs of RV overload. Even presented with normal systemic arterial pressure, these patients are at risk of deteriorating conditions and have worse prognosis [23,24]. Studies have shown that these patients may benefit from thrombolytic therapy [25,26]. The current guideline also suggests that thrombolytic therapy may be considered in selected patients with intermediate risk PE after thorough consideration of conditions increasing the risk of bleeding[3,27]. In this study, we therefore included these patients.

The subgroup analysis in our present paper was not pre-defined in the protocol. As a result, we did not have enough power to draw the conclusive data. Although this inclusion may potentially make our efficacy comparison of the two regimens less conclusive, subgroup analysis did show that UK-2 h regimen was effective not only for patients with intermediate risk PE but also for patients with hemodynamic instability. Nevertheless, future studies with anticoagulation control in patients with intermediate risk PE will be valuable to determine whether thrombolytic therapy is indeed beneficial in these patients. Currently, giving LMWH is not a routine practice for high risk PE patients. However, subcutaneous injection of LMWH has been shown to produce greater efficacy, less hemorrhagic risk, and improved convenience in clinical research and practice. Considering its advantages, it would be safe to use LMWH in high risk PE patients after thrombolytic therapy.

### Adverse events

The mortality rates in our study (1.4-3.4%) were comparable to those reported in previous clinical trials. Although 2 (3.4%) patients in the UK-2 h group died, both these two patients died in the early stage due to the severity of disease it self and commodities. The patient's death in the UK-12 h group was due recurrent PE.

The major bleeding rate in our study (5.7-10.2%) was comparable to previously reported trials [19,27,28] and the overall bleeding rate (13.6-14.3%) in our patients was lower than that in the UKEP study (24-29%)[29]. The high incidence of hemorrhagic complications observed in early studies was mainly attributed to the invasive procedures for angiographic and hemodynamic measurements [19,27,29]. No allergic reactions were observed in our study.

One major criteria in evaluating thrombolytic therapy is recurrence of embolism, most of which occur during the first week of follow-up and can result in a high mortality rate. The recurrence in our study was similarly low in both groups, with one patient in the UK-12 h group died of recurrent PE at day 4 after initial treatment. No recurrent PE was reported in UK-2 h group. Early thrombolytic therapy may reduce the incidence of recurrence of embolism. Further studies with prolonged observation are desirable to compare the recurrence between these two different regimens.

### Study limitations

There are a few limitations in our study. Firstly, the smaller sample size, a common limitation for PE thrombolytic studies, had prevented us from achieving more powerful efficacy and safety analysis. This is particularly true for smaller sample size for subgroup comparisons of efficacy and the adverse events between the two treatments in hemodynamically and anatomically massive PE subgroups. Therefore, we could not draw a confirmed conclusion. Further studies with larger sample size are desirable to determine the efficacy and safety of this UK 2 h regimen. There is a serious imbalance in the number of patients between the two treatment groups which was attributable to smaller case numbers assigned to 2-hour vs 12-hour UK infusion. Larger sample size and centralized randomization should avoid this type of imbalance to some extent.

Secondly, for the patients with extensive pulmonary artery obstruction and RV dysfunction, a control group with heparin alone was not included. Future studies with anticoagulation control will be valuable to determine whether thrombolytic therapy is indeed beneficial in these patients. The PEITHO (Pulmonary EmbolIsm THrOmbolysis) study [30] is on going to determine whether thrombolysis can improve the outcome of patients presenting with high-risk submassive acute PE compared with anticoagulant alone, which will demonstrate the clinical benefits of tenecteplase over placebo in normotensive patients with acute PE and right ventricular dysfunction. Few randomized trials have assessed the potential long-term benefits of thrombolytic therapy in acute massive PE except the study reported by Meneveau et al [31]. The study suggested that a 2-h regimen of streptokinase can be routinely used in patients with massive PE and maintained cardiac output without obviously compromising efficacy or safety. The long-term follow-up study would provide more valuable information on thrombolytic therapy.

## Conclusions

In conclusion, our study demonstrated that UK-2 h (20 000 IU/Kg) regimen displayed similar efficacy and safety as the UK-12 h regimen in treating acute PE with either hemodynamic instability or massive pulmonary artery obstruction. Given the convenience, lower cost and the similar efficacy and safety as the UK-12 h regime, we suggest that body weight adjusted UK-2 h regimen could be used for PE treatment. Further studies are required to evaluate the short and long-term outcomes of these different UK thrombolytic regimens.

## List of abbreviations

ACCP: American College of Chest Physician; CTPA: computed tomographic pulmonary angiography; DVT: deep vein thrombosis; ICH: intracranial hemorrhage; INR: international normalized ratio; LMWH: low molecular weight heparin; PE: pulmonary embolism; RVD: right ventricular dysfunction; RVED/LVED: right and left ventricular end-diastolic diameter ratio in the parasternal long-axis view; SPAP: systolic pulmonary arterial pressure; UK: urokinase; V/Q: ventilation perfusion lung scan.

## Competing interests

The authors declare that they have no competing interests.

## Authors' contributions

All authors made substantial contributions to conception and design, or acquisition of data, or analysis and interpretation of data; reviewed and approved the final manuscript; and contribute significantly to this study. Drs CW, ZGZ, and YHY contributed equality to the work. CW, the principal investigator, takes full responsibility for the integrity of the submission and publication, and was involved in study design as part of the Steering committee. ZGZ, had full access to all the data in the study and takes responsibility for the integrity of the data and the accuracy of the data analysis, and was responsible for the data verification, analysis and draft of the manuscript. YHY, had full access to all the data in the study and takes responsibility for the integrity of the data and the accuracy of the data analysis. YDY, was responsible for the patient enrollment and the data collection. ZZC, was responsible for the patient enrollment and the data collection. LRL, had full access to all the data in the study and takes responsibility for the integrity of the data and the accuracy of the data analysis. HPD, was responsible for the patient enrollment and the data collection. KWH, was responsible for the patient enrollment and the data collection. WXL, was involved in study design as part of the Steering committee. ZHZ was involved in study design as part of the Steering committee. XSC was involved in study design as part of the Steering committee. YHS was responsible for the data verification, analysis and draft of the manuscript. The contribution of the whole team in China Venous Thromboembolism Study Group was crucial to the success of this study.

Other contributions: The authors are also grateful to Drs. Charles A. Hales and Lan Zhao for their valuable suggestions on this manuscript.

## References

[B1] Cella G Palla A Sasahara AA Controversies of different regimens of thrombolytic therapy in acute pulmonary embolism Semin Thromb Hemost 1987 13 163 70 10.1055/s-2007-1003488 3306927

[B2] Gupta S Gupta BM Acute pulmonary embolism advances in treatment J Assoc Physicians India 2008 56 185 91 18697636

[B3] Kearon C Kahn SR Agnelli G Goldhaber S Raskob GE Comerota AJ Antithrombotic therapy for venous thromboembolic disease: American College of Chest Physicians Evidence-Based Clinical Practice Guidelines Chest 2008 133 8 454S 545S 10.1378/chest.08-0658 18574272

[B4] Kuthan F Urokinase pulmonary embolism trial JAMA 1971 215 1503 10.1001/jama.215.9.1503 5107636

[B5] Wenger NK Pulmonary embolism and the urokinase pulmonary embolism trial J Med Assoc Gay 1970 59 161 2 4913512

[B6] Wang F Wang C Wang T Pang BS Wu YB Yang YH Li C Zhang HY Weng XZ [Experimental study of the thrombolytic effects in a canine model of pulmonary thromboembolism induced by autologous radioactive blood clots] Zhonghua Jie He He Hu Xi Za Zhi 2004 27 93 6 14990182

[B7] Tebbe U Graf A Kamke W Zahn R Forycki F Kratzsch G Berg G Hemodynamic effects of double bolus reteplase versus alteplase infusion in massive pulmonary embolism Am Heart J 1999 138 39 44 10.1016/S0002-8703(99)70243-7 10385761

[B8] Fava M Loyola S Flores P Huete I Mechanical fragmentation and pharmacologic thrombolysis in massive pulmonary embolism J Vasc Interv Radiol 1997 8 261 6 10.1016/S1051-0443(97)70552-9 9083994

[B9] Ashton RW Daniels CE Ryu JH Thrombolytic therapy in patients with submassive pulmonary embolism N Engl J Med 2003 348 357 9 10.1056/NEJM200301233480416 12540653

[B10] Kreit JW The impact of right ventricular dysfunction on the prognosis and therapy of normotensive patients with pulmonary embolism Chest 2004 125 1539 45 10.1378/chest.125.4.1539 15078772

[B11] Zhu L Yang YH Wu YF Zhai ZG Wang C Value of transthoracic echocardiography combined with cardiac troponin I in risk stratification in acute pulmonary thromboembolism Chin Med J (Engl) 2007 120 17 21 17254482

[B12] Stein PD Terrin ML Gottschalk A Alavi A Henry JW Value of ventilation/perfusion scans versus perfusion scans alone in acute pulmonary embolism Am J Cardiol 1992 69 1239 41 10.1016/0002-9149(92)90944-T 1575198

[B13] Meyer G Collignon MA Guinet F Jeffrey AA Barritault L Sors H Comparison of perfusion lung scanning and angiography in the estimation of vascular obstruction in acute pulmonary embolism Eur J Nucl Med 1990 17 315 9 10.1007/BF01268022 2126770

[B14] Qanadli SD El M Hajjam Vieillard-Baron A Joseph T Mesurolle B Oliva VL Barré O Bruckert F Dubourg O Lacombe P New CT index to quantify arterial obstruction in pulmonary embolism: comparison with angiographic index and echocardiography AJR Am J Roentgenol 2001 176 1415 20 11373204 10.2214/ajr.176.6.1761415

[B15] Meer RW van der Pattynama PM van Strijen MJ Berg-Huijsmans AA van den Hartmann IJ Putter H de Roos A Huisman MV Right ventricular dysfunction and pulmonary obstruction index at helical CT: prediction of clinical outcome during 3-month follow-up in patients with acute pulmonary embolism Radiology 2005 235 798 803 10.1148/radiol.2353040593 15845793

[B16] Sasahra AA Bell WR Simon TL Stengle JM Sherry S The phase II urokinase-streptokinase pulmonary embolism trial: a national cooperative study Thromb Diath Haemorrh 1975 33 464 76 1098216

[B17] Walsh PN Stengle JM Sherry S The urokinase-pulmonary embolism trial Circulation 1969 39 153 6 4884574 10.1161/01.cir.39.2.153

[B18] Meyer G Sors H Charbonnier B Kasper W Bassand JP Kerr IH Lesaffre E Vanhove P Verstraete M Effects of intravenous urokinase versus alteplase on total pulmonary resistance in acute massive pulmonary embolism: a European multicenter double-blind trial. The European Cooperative Study Group for Pulmonary Embolism J Am Coll Cardiol 1992 19 239 45 1732347 10.1016/0735-1097(92)90472-y

[B19] Goldhaber SZ Kessler CM Heit J Markis J Sharma GV Dawley D Nagel JS Meyerovitz M Kim D Vaughan DE Randomised controlled trial of recombinant tissue plasminogen activator versus urokinase in the treatment of acute pulmonary embolism Lancet 1988 2 293 8 10.1016/S0140-6736(88)92354-9 2899718

[B20] Cheng X He J Gao M Chen G Li S Zhang Z Zhao M Zhou S Zhao J Cheng L Zhang Z Chen Y Xiong C [Multicenter clinical trial on the efficacy of thrombolytic therapy with urokinase and/or anticoagulant with low molecular weight heparin in acute pulmonary embolism] Zhonghua Nei Ke Za Zhi 2002 41 6 10 11940288

[B21] Miller GA Sutton GC Acute massive pulmonary embolism. Clinical and haemodynamic findings in 23 patients studied by cardiac catheterization and pulmonary arteriography Br Heart J 1970 32 518 23 10.1136/hrt.32.4.518 5433313 PMC487364

[B22] Tow DE Simon AL Comparison of lung scanning and pulmonary angiography in the detection and follow-up of pulmonary embolism: the Urokinase-Pulmonary Embolism Trial experience Prog Cardiovasc Dis 1975 17 239 45 10.1016/S0033-0620(75)80015-6 1114271

[B23] Lim KE Chan CY Chu PH Hsu YY Hsu WC Right ventricular dysfunction secondary to acute massive pulmonary embolism detected by helical computed tomography pulmonary angiography Clin Imaging 2005 29 16 21 10.1016/j.clinimag.2004.04.023 15859013

[B24] Konstantinides S Geibel A Heusel G Heinrich F Kasper W Heparin plus alteplase compared with heparin alone in patients with submassive pulmonary embolism N Engl J Med 2002 347 1143 50 10.1056/NEJMoa021274 12374874

[B25] Ashton RW Daniels CE Ryu JH Thrombolytic therapy in patients with submassive pulmonary embolism N Engl J Med 2003 348 357 9 10.1056/NEJM200301233480416 12540653

[B26] Torbicki A Perrier A Konstantinides S Agnelli G Galiè N Pruszczyk P Bengel F Brady AJ Ferreira D Janssens U Klepetko W Mayer E Remy-Jardin M Bassand JP Vahanian A Camm J De Caterina R Dean V Dickstein K Filippatos G Funck-Brentano C Hellemans I Kristensen SD McGregor K Sechtem U Silber S Tendera M Widimsky P Zamorano JL Zamorano JL Andreotti F Ascherman M Athanassopoulos G De Sutter J Fitzmaurice D Forster T Heras M Jondeau G Kjeldsen K Knuuti J Lang I Lenzen M Lopez-Sendon J Nihoyannopoulos P Perez L Isla Schwehr U Torraca L Vachiery JL Task Force for the Diagnosis and Management of Acute Pulmonary Embolism of the European Society of Cardiology Guidelines on the diagnosis and management of acute pulmonary embolism: the Task Force for the Diagnosis and Management of Acute Pulmonary Embolism of the European Society of Cardiology (ESC) Eur Heart J 2008 29 2276 315 10.1093/eurheartj/ehn475 18757870

[B27] Kanter DS Mikkola KM Patel SR Parker JA Goldhaber SZ Thrombolytic therapy for pulmonary embolism. Frequency of intracranial hemorrhage and associated risk factors Chest 1997 111 1241 5 10.1378/chest.111.5.1241 9149576

[B28] Terrin M Goldhaber SZ Thompson B Selection of patients with acute pulmonary embolism for thrombolytic therapy. Thrombolysis in pulmonary embolism (TIPE) patient survey. The TIPE Investigators Chest 1989 95 279S 81S 2495912

[B29] The UKEP study: multicentre clinical trial on two local regimens of urokinase in massive pulmonary embolism. The UKEP Study Research Group Eur Heart J 1987 8 2 10 3545842

[B30] PEITHO Pulmonary Embolism Thrombolysis Study http://clinicaltrials.gov/ct2/show/NCT00639743

[B31] Meneveau N Schiele F Metz D Valette B Attali P Vuillemenot A Grollier G Elaerts J Mossard JM Viel JF Bassand JP Comparative efficacy of a two-hour regimen of streptokinase versus alteplase in acute massive pulmonary embolism: immediate clinical and hemodynamic outcome and one-year follow-up J Am Coll Cardiol 1998 31 1057 63 10.1016/S0735-1097(98)00068-0 9562007

